# Evaluation and Comparison of Interocclusal Registration Accuracy Using Digital Intraoral Scanners and Conventional Methods: An In Vivo Study

**DOI:** 10.7759/cureus.89266

**Published:** 2025-08-02

**Authors:** Chiramana Sandeep, Shahista Afreen, Vijay B Kumar, Pallagani Lakshmi N Srujana, Sadvi Guntupalli, Sowrabha Adavikatla

**Affiliations:** 1 Department of Prosthodontics, Sibar Institute of Dental Sciences, Guntur, IND

**Keywords:** bite registration, intraoral, polysiloxane, scanners, wax

## Abstract

Introduction: This study aimed to assess and compare the precision of interocclusal registration using digital intraoral scanners and conventional materials. Specifically, it evaluated the accuracy of two commercially available intraoral scanners, examined the precision of two conventional interocclusal registration materials, and compared their outcomes to determine their relative effectiveness in clinical practice.

Materials and methods: This in vivo study was conducted in the Department of Prosthodontics on 12 patients with Angle’s Class I occlusion, who were divided into four groups based on the technique used for interocclusal registration. Group A used the 3Shape Trios 3 intraoral scanner (3Shape, Copenhagen, Denmark), Group B used the Medit i500 intraoral scanner (Medit, Seoul, South Korea), Group C used Jet Bite polyvinyl siloxane bite‑registration material (Coltene Whaledent, Altstätten, Switzerland; Batch no. 5171123100), and Group D used Aluwax bite‑registration wax (Maarc, Mumbai, India; Batch no. 2202/01/062022). Conventional impressions were made using GC Flexceed polyvinyl siloxane (GC Corporation, Tokyo, Japan; Batch no. 2305221) in perforated dentulous stock trays (Jabbar Company, India). The casts were fabricated using Type IV gypsum (Zooenta, Neelkanth HealthCare Pvt. Ltd., Jodhpur, India) and scanned using a Dentium Rainbow laboratory scanner (Dentium, Seoul, South Korea). Digital and conventional datasets were superimposed using a best‑fit alignment algorithm in three‑dimensional (3D) evaluation software to assess discrepancies. Statistical analyses were performed using repeated‑measures analysis of variance (ANOVA) and post hoc Bonferroni test (p < 0.05).

Results: Significant differences were observed among the four methods (p = 0.001). The 3Shape Trios 3 scanner showed the lowest discrepancy, followed by the Medit i500 and polyvinyl siloxane. Bite‑registration wax demonstrated the greatest discrepancy. Post hoc analysis confirmed significant differences between wax and the other methods, with no notable differences among the digital scanners and polyvinyl siloxane.

Conclusion: Digital intraoral scanners offer superior precision in interocclusal registration compared to bite‑registration wax, with an accuracy comparable to that of polyvinyl siloxane. These findings support the adoption of digital scanners in clinical practice to enhance restorative outcomes, although polyvinyl siloxane remains a reliable alternative.

## Introduction

In prosthetic and restorative dentistry, achieving accurate interocclusal registration is paramount to ensuring the functional and esthetic success of dental restorations [1]. Interocclusal registration refers to the process of recording the positional relationship between the maxillary and mandibular arches and is critical for fabricating restorations that align harmoniously with a patient’s occlusal dynamics [2]. Traditionally, this process relies on conventional techniques, such as wax, silicone, or polyvinyl siloxane, to capture the occlusal relationship [1]. These widely used methods are susceptible to challenges, including material distortion, operator variability, and patient discomfort, which can compromise the precision of the records and, consequently, the quality of the final restoration [3]. In recent years, advancements in digital dentistry have introduced innovative tools, such as intraoral scanners and computer‑aided design/computer‑aided manufacturing (CAD/CAM) systems, which promise enhanced precision, efficiency, and reproducibility in interocclusal registration [1,3]. These digital techniques aim to overcome the limitations of conventional methods by providing a more streamlined and accurate workflow; however, their clinical efficacy and precision compared with traditional approaches remain areas of active investigation [4].

The shift toward digital dentistry reflects broader technological advancements in healthcare, where precision, predictability, and patient‑centered outcomes are increasingly prioritized. For instance, intraoral scanners capture three‑dimensional (3D) images of dental arches, allowing digital interocclusal records to be integrated into virtual articulators for treatment planning [5]. These systems eliminate the need for physical impression materials, thereby reducing errors caused by material shrinkage, improper handling, or patient movement during the impression process. Furthermore, digital records can be stored indefinitely, manipulated for analysis, and seamlessly integrated into CAD/CAM workflows for restoration fabrication [5]. However, despite these advantages, digital techniques pose challenges. Scanner accuracy, software algorithms, and operator expertise can all influence the quality of digital interocclusal records [6,7]. Additionally, the high cost of digital equipment and the learning curve associated with its adoption may limit accessibility in certain clinical settings [8].

Given the critical role of interocclusal registration in achieving optimal occlusal harmony, it is essential to evaluate the precision of both digital and conventional techniques [1,3]. Previous studies have suggested that digital methods may offer superior accuracy by minimizing human error and material‑related discrepancies [1,3,4]. A systematic review by Nagri et al. [4] compared the accuracy of conventional and digital interocclusal records and included five studies. The authors reported high bias and heterogeneity among the studies. Moreover, in vivo studies that account for clinical variables, such as saliva, soft tissue interference, and patient cooperation, are necessary to validate these findings in real‑world scenarios. Such studies provide a more comprehensive understanding of how these techniques perform under practical conditions, where patient comfort, time efficiency, and cost‑effectiveness influence clinical decision‑making.

Therefore, this study aimed to assess and compare the precision of interocclusal registration achieved using digital and conventional techniques. The objectives were to evaluate the accuracy of interocclusal registration using two commercially available intraoral scanners, examine the precision of registrations performed with two conventional interocclusal registration materials, and compare the accuracy outcomes between digital and conventional methods to determine their relative effectiveness in clinical practice.

## Materials and methods

Study design and setting

This in vivo study was conducted at the Department of Prosthodontics, Sibar Institute of Dental Sciences, Guntur, India, over 12 months, from January 2024 to December 2024. This study employed a comparative experimental design to evaluate and compare the precision of interocclusal registration using digital and conventional techniques. Ethical clearance was obtained from the Institutional Ethics Committee (Pr.189/IEC/SIBAR/2023) in accordance with the principles of the Declaration of Helsinki. Informed consent was obtained from all patients prior to the study, detailing the procedures and their involvement in the study.

Sample size

The sample size was determined using the G*Power software (version 3.1.9.2, Heinrich-Heine-Universität Düsseldorf, Düsseldorf, Germany), with an estimated effect size of 0.72 between the conventional and digital bite-registration accuracy, derived from a previous study [3]. The study required a total of 12 patients to achieve a statistical power of 80% and an alpha error of 0.5%.

Eligibility criteria

Patients aged 18-25 years with skeletal Angle’s Class I occlusion, without dental rehabilitation, and exhibiting good oral hygiene, as assessed using the Oral Hygiene Index [9], were included. The exclusion criteria were as follows: patients who underwent restorative procedures, had periodontal conditions, underwent orthodontic extractions, received ongoing dental treatments, had temporomandibular joint disorders, or were unwilling to provide consent. All patients underwent oral prophylaxis to meet the inclusion criteria before enrolment.

Methodology

**Figure 1:**
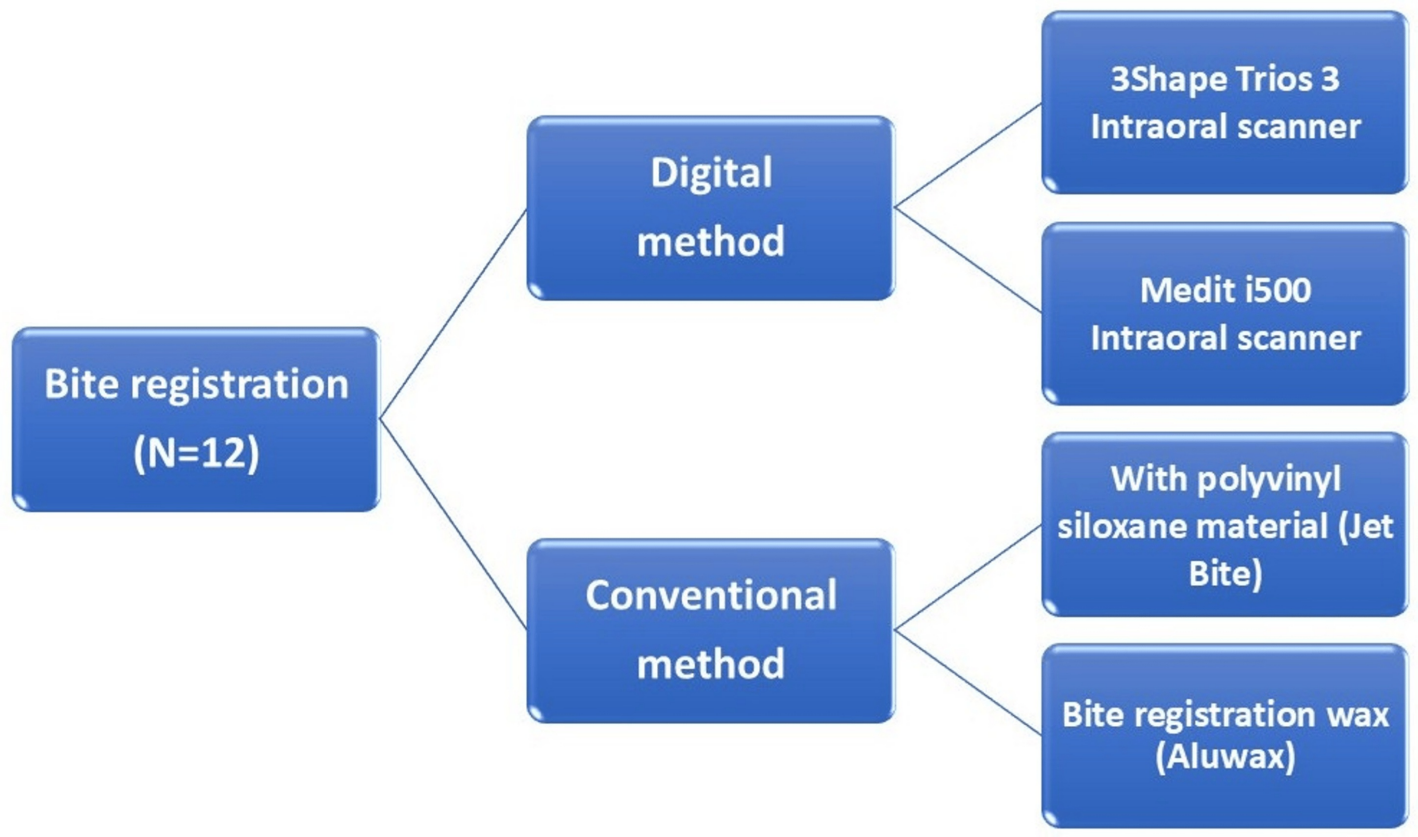
Study flowchart. Group A denotes 3Shape Trios 3 intraoral scanner; Group B denotes Medit i500 intraoral scanner; Group C denotes polyvinyl siloxane as Jet Bite; Group D denotes bite-registration wax as Aluwax; N denotes number of patients.

The study evaluated interocclusal registrations in 12 patients using two digital intraoral scanners and two conventional materials (Figure 1), with the following samples: Group A used the 3Shape Trios 3 intraoral scanner (3Shape, Copenhagen, Denmark); Group B used the Medit i500 intraoral scanner (Medit, Seoul, South Korea); Group C used polyvinyl siloxane bite-registration material (Jet Bite, Coltene Whaledent, Altstätten, Switzerland, Batch no. 5171123100); and Group D used bite-registration wax (Aluwax, Maarc, Mumbai, India, Batch no. 2202/01/062022).

**Figure 2:**
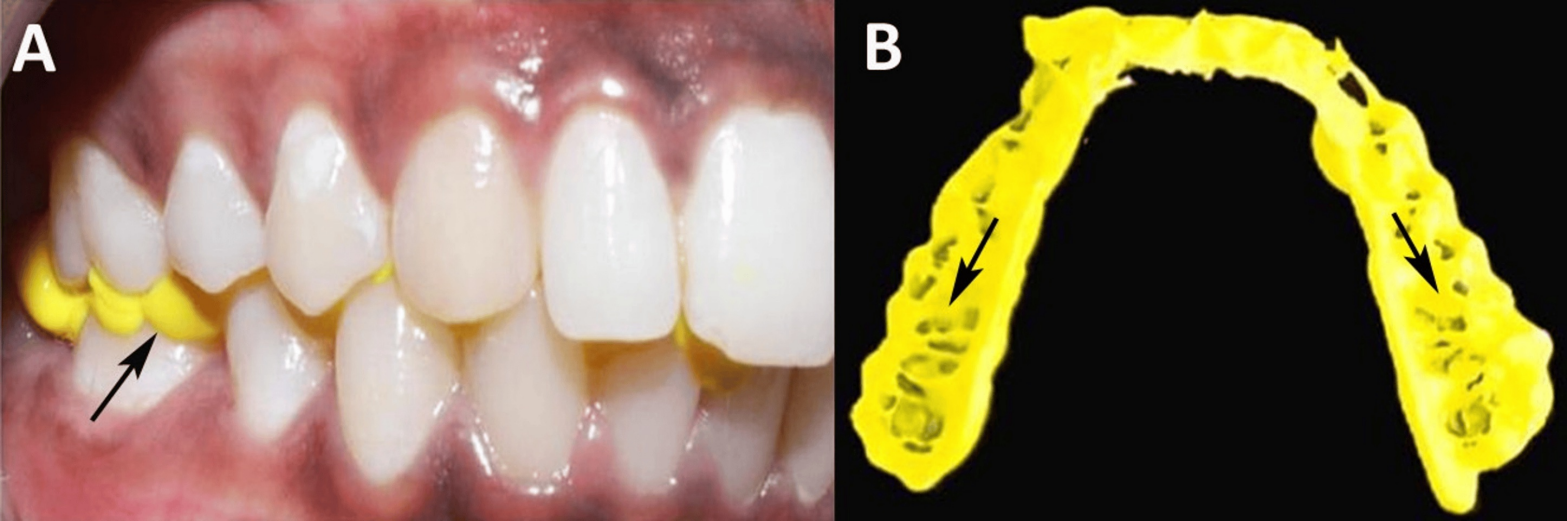
Bite registration with Jet Bite. (A) Material application for bite registration. (B) Bite registration illustrating the accurate alignment and contact points of the teeth. Original image of a patient from the study, and used with patient's consent for publication.

Conventional impressions were made using polyvinyl siloxane (GC Flexceed Kit, GC Corporation, Tokyo, Japan; Batch no. 2305221) in perforated dentulous stock trays (Jabbar Company, India) using a dual-step technique. A polyethylene sheet was used to separate the putty and light-body layers to capture detailed maxillary and mandibular impressions, which were disinfected with 1.25% sodium hypochlorite solution (Maxsol, India) for 5 minutes, rinsed, dried, and stored in sealed zip-lock bags. Bite registrations for Group C involved dispensing Jet Bite using an automatic dispensing gun (Coltene Whaledent, Altstätten, Switzerland), with patients biting into centric relations to capture their occlusal relationships (Figure 2).

**Figure 3:**
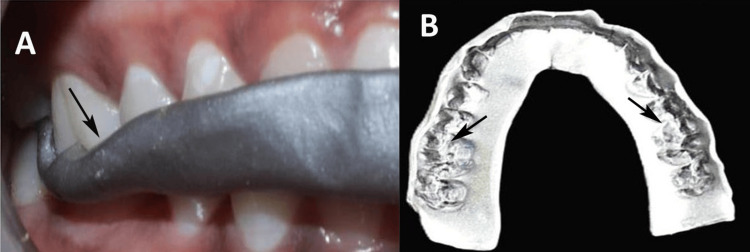
Bite registration with Aluwax. (A) Material application for bite registration. (B) Bite registration illustrating the accurate alignment and contact points of the teeth. Original image of a patient from the study, and used with patient's consent for publication.

The set material was examined using a magnifying glass, disinfected, and then stored in airtight containers. In Group D, Aluwax was softened, shaped into a 2 mm thick arch, placed over the maxillary teeth, and bitten into occlusion (Figure 3).

**Figure 4:**
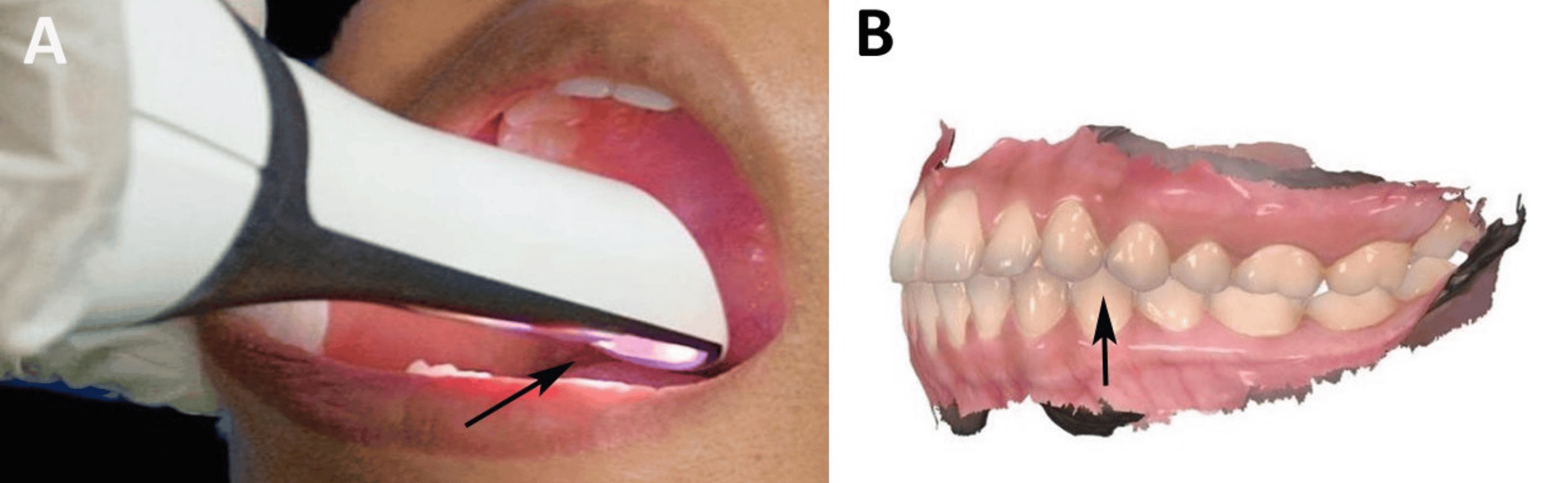
Digital scan. (A) Capturing a digital three-dimensional (3D) scan of the patient’s oral cavity. (B) 3D visualization from the intraoral scan. Original image of a patient from the study, and used with patient's consent for publication.

The wax was inspected, disinfected, rinsed, and stored in water-filled containers to prevent distortion. Type IV gypsum (Zooenta, Neelkanth HealthCare Pvt. Ltd., Jodhpur, India) was used to fabricate the casts, which were hand-articulated with Jet Bite or Aluwax records and secured with rubber bands. The casts were individually and in occlusion scanned using a Dentium rainbow laboratory scanner (Dentium, Seoul, South Korea), and the data were converted to the Standard Tesselation Language (STL) format for three-dimensional (3D) analysis. Digital scans of Groups A and B captured the maxillary and mandibular arches and occlusal surfaces, with soft tissue data trimmed to focus on the hard tissues (Figure 4).

**Figure 5:**
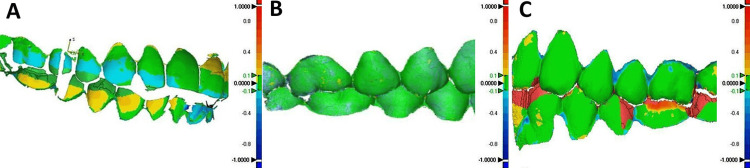
Discrepancy analysis using best-fit algorithm: (A) between conventional methods, (B) between digital methods, and (C) between conventional and digital methods. Colorbar denotes absolute discrepancies in millimeters (mm). These deviations indicate the degree of mismatch between two interocclusal registration methods. Warmer colors (such as red or orange) correspond to higher deviations, while cooler colors (such as blue or green) reflect closer alignment and thus better accuracy. Original image of the data from the study.

STL datasets from both digital and conventional methods were superimposed using a best-fit alignment algorithm in 3D evaluation software to compare the precision and identify the contact points and discrepancies. The best-fit algorithm was used to ascertain the contact points of each dataset relative to its respective counterpart. The alignment elucidated the regions where the datasets intersected and delineated the exact contact points between them. The absolute values of these contact points were subsequently extracted and averaged to evaluate the degree of concordance between the repeated measurements (Figure 5). This methodology offers a robust framework for assessing the variance between disparate measurements, thereby providing an objective evaluation of data precision. The overall precision of interocclusal registration derived from various methodologies was appraised by analyzing average absolute values.

Calibration and reliability

To ensure consistency, all procedures were performed by a single trained operator, who was calibrated through pilot testing on five non-study patients. The operator was trained to standardize the tray placement, material mixing, and scanning protocols. The intraoral scanners were calibrated according to the manufacturer’s guidelines before each session. The reliability of conventional bite registration was verified by examining the set materials under a magnifying glass and repeating the procedures if discrepancies were found. Digital scans were checked for completeness and artifacts, and rescanning was performed if necessary. The accuracy of the laboratory scanner was validated by scanning a reference model before the study to ensure reliable data capture.

Statistical analysis

Data were analyzed using IBM SPSS Statistics for Windows, Version 20 (Released 2011; IBM Corp., Armonk, New York, United States). The differences in interocclusal registration between the digital and conventional methods were measured and expressed as means and standard deviations. The normality of the data was assessed using the Shapiro-Wilk test. Intergroup (within-subject design) comparisons were performed using repeated analysis of variance (ANOVA), followed by post-hoc Bonferroni test for pairwise comparisons, as the data were parametric. Statistical significance was set at p < 0.05.

## Results

Demographic details of study population are presented in Table 1.

**Table 1:** Demographic details of the study participants.

Parameters	Category	Values
Age, years (mean ± SD)		26.34 ± 4.65
Sex	Male (n (%))	6 (50%)
	Female (n (%))	6 (50%)

Intergroup comparisons of discrepancies in interocclusal registration revealed statistically significant differences among the four methods (p = 0.001). The lowest mean discrepancy was observed with the 3Shape Trios 3 intraoral scanner (0.01 ± 0.05 mm), followed by the Medit i500 intraoral scanner (0.04 ± 0.14 mm) and polyvinyl siloxane bite-registration (0.08 ± 0.18 mm) methods. In contrast, the bite-registration wax group exhibited the highest discrepancy (0.39 ± 0.25 mm). The significant p-value (p < 0.05) indicated that digital scanners provided more accurate interocclusal registrations than conventional methods. These findings suggest that digital impression systems may offer superior precision in recording occlusal relationships, potentially reducing clinical errors in prosthetic and restorative workflows (Table 2).

**Table 2:** Mean discrepancy (in mm) across different interocclusal registration methods with intergroup comparison using repeated ANOVA in a sample of 12 patients (within-subjects). *p < 0.05 denotes statistical significance using repeated analysis of variance (ANOVA) test. The table shows the mean and standard deviation of absolute discrepancies (in mm) for each study group. Discrepancy values represent the average deviation between repeated registrations per group, assessed using best-fit superimposition in three-dimensional evaluation software. Repeated ANOVA was used to compare discrepancies between groups (within-subjects).

Groups	n (%)	Mean	SD	F value	p-value
Group A (3Shape Trios 3 intraoral scanner)	12 (100)	0.01	0.05	12.66	0.001*
Group B (Medit i500 intraoral scanner)	12 (100)	0.04	0.14		
Group C (Polyvinyl siloxane bite-registration material)	12 (100)	0.08	0.18		
Group D (Bite-registration wax)	12 (100)	0.39	0.25		

The Bonferroni test revealed significant differences in interocclusal registration discrepancies between specific groups. The largest mean difference was observed between the 3Shape Trios 3 intraoral scanner and bite-registration wax (0.38 mm, p = 0.0001), followed by the Medit i500 intraoral scanner and bite-registration wax (0.35 mm, p = 0.0001) and bite-registration wax and polyvinyl siloxane bite-registration material (0.31 mm, p = 0.0003). In contrast, no significant differences were found between the Medit i500 intraoral scanner and 3Shape Trios 3 intraoral scanner (p = 0.9731), Medit i500 intraoral scanner and polyvinyl siloxane bite-registration material (p = 0.9396), or 3Shape Trios 3 intraoral scanner and polyvinyl siloxane bite-registration material (p = 0.7487). These results indicated that bite-registration wax, a conventional method, produced significantly greater discrepancies compared to both digital scanners and other conventional methods (polyvinyl siloxane bite-registration material). The minimal differences between digital systems and polyvinyl siloxane bite-registration material suggested comparable accuracy, whereas bite-registration wax appeared less reliable for precise interocclusal registration. Clinically, digital scanners may be preferable to improve the accuracy of prosthetic and restorative procedures (Table 3).

**Table 3:** Pairwise comparison of mean discrepancies (in mm) between interocclusal registration methods using the post-hoc Bonferroni test. *p < 0.05 denotes statistical significance using the Bonferroni test, indicating a meaningful difference in accuracy between methods. **Mean difference in bite registration discrepancy (in mm) between each pair of methods.

Pairwise groups		Mean difference**	CI at 95%		T stat	p-value
			Lower limit	Upper limit		
Medit i500 intraoral scanner	3Shape Trios 3 intraoral scanner	0.03	-0.2164	0.1564	0.041	0.9731
Medit i500 intraoral scanner	Bite-registration wax	-0.35	0.1636	0.5364	4.231	0.0001*
Medit i500 intraoral scanner	Polyvinyl siloxane bite-registration material	-0.04	-0.1464	0.2264	0.023	0.9396
3Shape Trios 3 intraoral scanner	Bite registration-wax	-0.38	0.1936	0.5664	3.761	0.0001*
3Shape Trios 3 intraoral scanner	Polyvinyl siloxane bite-registration material	-0.07	-0.1164	0.2564	0.186	0.7487
Bite-registration wax	Polyvinyl siloxane bite-registration material	0.31	-0.4964	-0.1236	3.192	0.0003*

## Discussion

Intergroup comparison of interocclusal registration methods highlighted significant differences in accuracy between digital intraoral scanners (3Shape Trios 3 and Medit i500), polyvinyl siloxane bite‑registration material, and bite‑registration wax. The superior performance of digital scanners, particularly the 3Shape Trios 3, underscores their potential to enhance the accuracy of prosthetic and restorative workflows.

The enhanced accuracy of digital intraoral scanners can be attributed to their ability to capture 3D data at high resolution with minimal distortion. Digital systems employ advanced optical technologies, such as lasers and structured light scanning, to record occlusal surfaces with precision [10]. Studies such as those by Mangano et al. [8] have demonstrated that intraoral scanners provide consistent and repeatable results for capturing dental anatomy compared with traditional methods. The minimal discrepancy observed with 3Shape Trios 3 and Medit i500 in this study supports these findings, suggesting that the digital workflow reduces errors associated with manual handling and material properties. Iwauchi et al. [1] and Ries et al. [3] have reported similar results. The lack of significant differences between the two scanners and polyvinyl siloxane bite‑registration material further indicates that digital systems may achieve accuracy comparable to well‑established conventional methods, such as polyvinyl siloxane, which is known for its dimensional stability and reliability [11]. In contrast, Ries et al. [3] reported better accuracy for the 3Shape Trios 3 intraoral scanner than for polyvinyl siloxane. This disparity may be due to differences in the evaluation methods used; they measured deviations in three separate axes, whereas average values were obtained in the present study.

The higher discrepancy observed with bite‑registration wax aligns with previous studies, highlighting its limitations [12,13]. Bite‑registration wax is susceptible to deformation under pressure, temperature changes, and improper handling, which can introduce occlusal registration errors [13]. These material properties likely contributed to the significant differences observed when compared with the digital scanners and polyvinyl siloxane. The lower rigidity and tendency of wax to distort during removal or storage may explain its inferior performance [12]. Dwivedi et al. [14] noted that wax‑based registration is less accurate than polyvinyl siloxane‑based bite registration. This finding underscores the challenges of relying on wax for accurate occlusal records, particularly in complex restorative cases in which precision is critical.

However, not all studies unequivocally favor the use of digital systems in dentistry. Some studies, such as that by Krahenbuhl et al. [15], reported statistically significant differences in the accuracy and precision of occlusal contacts among stereolithographic casts mounted using digital occlusal registrations, which were found to be inaccurate. This variation in results might be due to differences in scanner type. The absence of significant differences between polyvinyl siloxane and digital scanners in this study supports the perspective that polyvinyl siloxane remains a reliable option when digital systems are unavailable or impractical [11]. A previous study also reported better accuracy for polyvinyl siloxane impressions than for the Cerec Omnicam intraoral scanner [16].

Discrepancies among studies favoring conventional methods may arise from variations in operator skill, calibration of digital systems, or patient‑specific factors such as occlusal complexity [17]. Ensuring precision in intraoral scanning is a complex process influenced by various determinants. These include operator‑centric factors such as proficiency, experience, and ongoing education [6]. Additionally, patient‑related factors, including their level of cooperation, oral health status, and the characteristics and positioning of the scanned materials and preparations, can significantly influence the accuracy of scanning outcomes [18].

The discrepancies between studies that contrast with the current findings may also be related to technological differences among scanners. Not all intraoral scanners perform equally because their accuracy depends on the scanning technology, software algorithms, and calibration protocols [19]. Earlier studies have reported variability in scanner performance, particularly with older models that have lower resolution or less sophisticated software [6,18,19]. Winkler and Gkantidis [20] reported higher accuracy for the 3Shape Trios scanner than for the Carestream CS 3600 scanner.

Environmental and clinical factors may also contribute to discrepancies between studies. Digital scanners require a dry field and adequate lighting, and deviations from optimal conditions can affect scan quality [21]. In contrast, polyvinyl siloxane is less sensitive to such variables, which may explain why some studies have reported comparable or superior outcomes using conventional methods [11]. Additionally, the learning curve associated with digital scanners can influence results, as inexperienced operators may struggle to achieve optimal scans, leading to inconsistencies in study outcomes [18].

The significant discrepancy observed with bite‑registration wax compared with other methods highlights its limitations in modern dentistry. The susceptibility of the material to distortion and its inability to capture fine occlusal details make it less suitable for procedures requiring high precision, such as implant‑supported restorations and full‑mouth rehabilitation. These findings are consistent with previous studies [12,13].

Clinical implications

The superior accuracy of digital intraoral scanners suggests that their integration into routine dental practice can improve prosthetic and restorative dental outcomes. By minimizing discrepancies in interocclusal registration, digital systems can reduce the need for chairside adjustments, shorten treatment times, and enhance patient satisfaction. The comparable performance of polyvinyl siloxane indicates that it remains a viable option, particularly in practices where digital infrastructure is not yet available. However, the poor performance of bite-registration wax suggests that it should be used cautiously, if at all, in precision-critical cases. Clinicians adopting digital scanners should invest in training to optimize their use and ensure proper calibration to maintain the accuracy of the impressions. Future research should explore the long-term clinical outcomes of digital versus conventional methods and investigate the cost-effectiveness of transitioning to a digital workflow.

Limitations

This study had several limitations. First, although statistically determined, the sample size was relatively small (n = 12), which potentially limited the generalizability of our findings to the general population. Second, the study was conducted in a controlled setting with a single trained operator, which may not reflect real-world clinical variability, where operator experience and patient conditions differ from each other. Third, this study focused on patients with Angle’s Class I occlusion, limiting its applicability to other occlusal relationships in the population. Finally, environmental factors, such as salivary flow or patient movement during scanning, were not fully accounted for, which could influence the accuracy of digital scanning in clinical practice.

## Conclusions

This study demonstrated that digital intraoral scanners provided superior precision in interocclusal registration compared to conventional bite-registration wax, with accuracy comparable to that of polyvinyl siloxane. The significant discrepancies observed with wax highlight the limitations of precise occlusal recordkeeping. These findings support the adoption of digital scanners in prosthetic and restorative dentistry to enhance their accuracy and reduce clinical errors. However, polyvinyl siloxane remains a reliable alternative when digital systems are unavailable for making impressions. Clinicians should consider integrating digital workflows while ensuring proper training and calibration to maximize their precision.
